# Analyzing Occupational Safety Managers’ and Representatives’ Assessments of Collaboration With Occupational Health Care

**DOI:** 10.1177/21650799251392224

**Published:** 2025-12-20

**Authors:** Sari Nissinen, Anniina Kainalainen, Erja Sormunen

**Affiliations:** 1 Finnish Institute of Occupational Health

**Keywords:** occupational safety, occupational safety and health, occupational health care, collaboration, workload factors

## Abstract

**Background:**

Collaboration between occupational safety (OS) and occupational health care (OHC) is essential for workplace health and safety, yet the experiences of OS actors have been less studied.

**Objective:**

To examine OS managers’ and representatives’ experiences of collaboration with OHC and identify related factors.

**Methods:**

This cross-sectional study was conducted in Finland via an online survey in March–April 2025. A total of 222 OS managers and 364 OS representatives responded to a questionnaire. Data were analyzed using non-parametric methods. Group differences were examined with the Mann–Whitney *U* and Kruskal–Wallis tests. Spearman’s rank correlation was used to explore associations between background variables and attitude statements related to perceived seamless collaboration.

**Results:**

OS managers rated the collaboration as more seamless than OS representatives (mean 7.76 vs. 7.17; *p* < .001), and more often gave it an excellent rating (39% vs. 22%). Seamless collaboration was particularly associated with the OHC agreement’s comprehensiveness, OS experience, and digital tools. The strongest associations were found for OHC participation in OS activities (*r* = .78) and availability of workload data (*r* = .77).

**Conclusions:**

The availability of information related to workload factors and the participation of OHC in workplace OS activities are particularly important for a positive experience of collaboration. Seamless collaboration with OHC also requires clear, jointly agreed procedures.

**Application to Practice:**

OHC should strengthen its collaboration with OS by clearly identifying and communicating workload and resource factors in the work environment, in order to effectively and impactfully target development measures.

## Background

Identifying and assessing health hazards in the workplace, as well as monitoring the work environment and working methods to prevent risks to employee health, are central tasks of occupational safety (OS; International Labour Organization, 2020). However, OS practices vary between countries, even though their common goal is to ensure the safety and well-being of employees at work. The International Labour Organization (2020) requires that OS activities at workplaces be developed in collaboration with experts from various fields. The World Health Organization & International Labour Organization (2018) also emphasizes the importance of collaboration in effectively supporting workers’ health and safety. In addition, national strategies in different countries may emphasize various aspects, such as the prevention of specific work-related risks (European Agency for Safety and Health at Work, 2023).

The organization and operational models of occupational health care (OHC) also vary between countries (Rantanen et al., 2017). In Finland, employers are legally obligated to provide OHC services for their employees (Occupational Health Care Act 1383/2001, 2001). OHC activities are guided by good occupational health practice, which requires systematic and goal-oriented collaboration with the workplace. The primary objectives of OHC are to maintain employees’ health, prevent occupational diseases and accidents, and promote the health and safety of the work environment and work community. As part of this task, OHC conducts a workplace survey to assess the effects of work-related hazards and workload factors on employees’ health and work ability. In addition, OHC provides guidance on safe working methods and the use of protective equipment and offers recommendations for work development measures and harm prevention. In accordance with good practice, OHC utilizes the workplace’s own risk assessment of work-related hazards and workload factors in the workplace survey (Mattila, 2024).

The workplace survey forms the foundation for collaboration between the workplace and OHC and is a central part of OS activities. The information collected during the survey is compiled into a workplace survey report, which serves as the basis for planning OHC activities according to the needs of the workplace. The report is typically delivered to a designated contact person at the workplace, who is responsible for distributing it to management, supervisors, OS actors, and human resources. In accordance with good practice, OHC provides feedback on the survey to the entire staff and reviews the findings together with OS representatives (Halonen et al., 2017; Oksa et al., 2014).

The need to develop collaboration is particularly emphasized in the changing world of work, where rapid technological development is reshaping job content, work environments, and ways of working (European Agency for Safety and Health at Work, 2018). Digitalization and new technologies, such as artificial intelligence and robotics, bring both new opportunities and health and safety risks. Anticipating and managing these risks requires close collaboration between OS and OHC. At the same time, digitalization offers new means for collecting up-to-date, multi-source, and individualized information on working conditions and employee well-being. This creates a foundation for knowledge-based, effective occupational health and safety activities (Sallinen, 2024).

OHC plays a key role in promoting workplace health and safety. Workplaces especially expect OHC to take a proactive and preventive approach to managing workload factors in the work environment and to provide sufficient and clear communication regarding these factors (Halonen et al., 2017; Lydell et al., 2019; Majuri & Wallius, 2024). Active participation of OHC in the development of the work environment is particularly important, as the reduction of occupational diseases and work-related illnesses is often the result of functional and goal-oriented collaboration with OS (Reijula, 2022).

To date, there has been little research on collaboration between OS and OHC. Previous studies have mainly focused on general collaboration between workplaces and OHC (Halonen et al., 2017; Pesonen et al., 2019; Schmidt et al., 2015), the specific characteristics of collaboration in organizations of different sizes (Pesonen et al., 2025; Vinberg et al., 2017), as well as collaboration in promoting mental health (Majuri et al., 2024; Majuri & Wallius, 2024) and preventing musculoskeletal disorders (Sormunen et al., 2020). Based on these studies, the prerequisites for successful collaboration include jointly agreed goals and procedures, as well as mutual and up-to-date information exchange.

In Finland, workplace OS actors include the OS manager, who represents the employer, and one or more OS representatives, who represent the employees. These actors are jointly responsible for identifying and assessing hazards arising from work, working hours, workspaces, and other factors related to the work environment and conditions. They also collaborate with OHC to promote workplace safety and health. Previous research has not examined OS actors’ perspectives on collaboration with OHC. This study addresses that research gap by examining differences in OS managers’ and representatives’ views on collaboration with OHC and the factors associated with these views. The aim is to produce knowledge that supports the development of collaboration and the promotion of occupational health and safety in workplaces.

The research questions were:

Q1: How do occupational safety managers and representatives differ in their evaluations and experiences of collaboration with occupational health care?
Q2: Which factors are associated with perceived collaboration with occupational health care services?

## Material and Methods

### Study Design

This is a cross-sectional descriptive study conducted with data from OS actors.

### Participant Recruitment and Data Collection

The study was conducted as an online survey in Finland during March and April 2025. A sample of 2,000 OS managers and 2,000 OS representatives was drawn from the OS personnel register maintained by the Centre for Occupational Safety, which is administered by labor market organizations. The survey link was distributed to potential participants via email, followed by one reminder message. Some email addresses proved to be invalid, resulting in the survey reaching 1,517 OS managers and 1,848 OS representatives. Among the OS managers, 393 opened the survey link and 222 completed the survey (response rate 15%), while 726 OS representatives opened the link and 364 completed the survey (response rate 20%).

### Ethical Considerations

The study received ethical approval from the Ethics Committee of Finnish Institute of Occupational Health on February 25, 2025 (MFid 178271). The study followed the guidelines of the Finnish National Board on Research Integrity (TENK, 2023) and the principles of the Declaration of Helsinki. The data were accessible only to the research team, and individual respondents could not be identified.

Separate written consent was not requested from participants, as in Finland, responding to a survey is considered informed consent to participate in the study and to permit the use of the data. All participants received an official cover letter by email, which presented the background and purpose of the study and emphasized that participation was entirely voluntary and anonymous.

### Instrument

The questionnaire was developed specifically for this study, as there is limited prior research on the topic and no existing validated instruments were available. The questionnaire was designed by the research team, drawing on previous literature, the researchers’ own experience with OS and OHC collaboration, and recommendations related to occupational health and safety practices. The questionnaire included both structured statements and background variables, enabling diverse statistical analysis.

The questionnaire had nine structured items using a 4-point Likert scale, where 1 = Totally disagree, 2 = Somewhat disagree, 3 = Somewhat agree, and 4 = Totally agree for each item. Internal consistency was Cronbach’s α = .859 for OS managers and α = .845 for OS representatives. In addition to collaboration statements, the questionnaire included background variables (gender, age, education, work experience, work experience in occupational health, workplace size, occupational health/healthcare agreement; Table 1).

**Table 1.** Questionnaire Items and Measurement Scales Used to Assess Collaboration Between OS and OHC Professionals and Background Characteristics of Respondents

Questionnaire item	Recoded scale
*Statements of collaboration*	
Occupational health care is well aware of all working conditions at our workplace.	Agree 4 = Totally agree 3 = Somewhat agree Disagree 2 = Somewhat disagree 1 = Totally disagree
I have access to workload-related data when I need it.	Agreed 4 = Totally agree 3 = Somewhat agree Disagree 2 = Somewhat disagree 1 = Totally disagree
I have access to workload-related data in the format I need.	Agreed 4 = Totally agree 3 = Somewhat agree Disagree 2 = Somewhat disagree 1 = Totally disagree
The method of sharing workload-related data with occupational health care has been agreed upon.	Agreed 4 = Totally agree 3 = Somewhat agree Disagree 2 = Somewhat disagree 1 = Totally disagree
The method of sharing workload-related data with occupational health care should be clarified.	Agreed 4 = Totally agree 3 = Somewhat agree Disagree 2 = Somewhat disagree 1 = Totally disagree
Digital services or information systems are available for data exchange between occupational safety and occupational health care.	Agreed 4 = Totally agree 3 = Somewhat agree Disagree 2 = Somewhat disagree 1 = Totally disagree
Opportunities for occupational safety to collaborate with occupational health care should be increased.	Agreed 4 = Totally agree 3 = Somewhat agree Disagree 2 = Somewhat disagree 1 = Totally disagree
Occupational health care’s participation in our workplace’s occupational safety activities is sufficient.	Agreed 4 = Totally agree 3 = Somewhat agree Disagree 2 = Somewhat disagree 1 = Totally disagree
Overall, collaboration between our workplace and occupational health care is seamless.	Agreed 4 = Totally agree 3 = Somewhat agree Disagree 2 = Somewhat disagree 1 = Totally disagree
Rating of collaboration with occupational health care	Scale 0–10 (0 = very poor and 10 = excellent collaboration)
Gender	Male Female Other/prefer not to say
Age	<40 years 40–49 years 50–59 years ≥60 years
Education	Low education degree Basic/elementary school, vocational/technical/commercial school, upper secondary school, institute-level education (ISCED 1–4) High education degree University of applied sciences, university/higher education (bachelor’s, master’s, doctoral degrees) (ISCED 5–8)
Work experience	≤20 years >20 years
Occupational safety experience	<5 years 5–10 years >10 years
Working time allocated to occupational safety tasks	Full-time Part-time As needed
Workplace size	<50 employees 50–249 employees ≥250 employees
Industry sector of the workplace	Industry Health and social services Trade Public administration Construction Education Other Agriculture Forestry and fishing Mining and quarrying Electricity, gas, steam, and air conditioning supply Water supply, sewerage, waste management, and remediation Transportation and storage Accommodation and food service Financial and insurance Real estate Professional, scientific, and technical Administrative and support service Arts, sports, and recreation Other service
Occupational health care agreement	Preventive and medical services Preventive services only Don’t know Prefer not to say

*Note*. The ISCED classification is based on UNESCO’s International Standard Classification of Education (UNESCO Institute for Statistics, 2024). OS = occupational safety, OHC = occupational health care.

Additionally, respondents were asked to rate the overall collaboration with occupational health care on a numeric scale from 0 to 10. For reporting purposes, these responses were grouped into four categories: Poor (0–5), Moderate (6–7), Good (8), and Excellent (9–10). Details of the questionnaire items and measurement scales are presented in Table 1.

### Data Analysis

The data were analyzed using SPSS Statistics version 30. Descriptive statistics (frequencies and percentages) were used to summarize the data. Some variables were recoded to simplify the analysis and improve compatibility with statistical methods. Non-parametric tests were applied due to the non-normal distribution of variables. Group differences were examined using the chi-square test, Mann–Whitney *U* test, and Kruskal–Wallis test, depending on the number of comparison groups. Statistical significance of differences was assessed with the chi-square test, with a significance threshold of *p* < .05. A sum variable was created and treated as continuous in the analysis. Associations between continuous variables and a sum variable of seamless collaboration were assessed using Spearman’s rank correlation coefficient.

## Results

### Differences in Experiences of Collaboration

A total of 586 individuals responded to the survey, including 222 OS managers and 364 OS representatives. The mean age of OS managers was 51 years with an average of 29 years of total work experience and 11 years of experience in OS tasks. Among OS representatives, the mean age was 49 years, general work experience averaged 27 years, and OS experience averaged 7 years.

The results indicated statistically significant differences between OS managers and OS representatives in several background variables (Table 2). OS managers more frequently had a higher education degree (ISCED 5–8; 73% vs. 43%, *p* < .001) and more than 10 years of OS experience (44% vs. 18%, *p* < .001) compared to OS representatives.

**Table 2.** Demographic Characteristics of Occupational Safety Managers (*n* = 222) and Representatives (*n* = 364)

Variable	Occupational safety managers (*n* = 222), *n* (%)	Occupational safety representatives (*n* = 364), *n* (%)	***p***
*Gender*			.063
Male	112 (51)	155 (43)	
Female	102 (46)	195 (53)	
Other/prefer not to say	3 (1)	6 (2)	
Missing	5 (2)	8 (2)	
*Age*			.231
<40 years	26 (11)	47 (13)	
40–49 years	48 (22)	96 (27)	
50–59 years	86 (39)	111 (30)	
≥60 years	37 (17)	65 (18)	
Missing	25 (11)	45 (12)	
*Education*			<.001
Low education degree (ISCED 1–4)^a^	57 (26)	206 (57)	
High education degree (ISCED 5–8)^b^	162 (73)	156 (43)	
Other	1 (0)	0 (0)	
Missing	2 (1)	2 (0)	
*Work experience*			.004
≤20 years	49 (22)	120 (33)	
>20 years	172 (78)	241 (66)	
Missing	1 (0)	3 (1)	
*Occupational safety experience*			<.001
<5 years	57 (26)	147 (41)	
5–10 years	63 (28)	140 (38)	
>10 years	98 (44)	67 (18)	
Missing	4 (2)	10 (3)	
*Working time allocated to OS tasks*			.015
Full-time	33 (15)	76 (21)	
Part-time	136 (62)	230 (63)	
As needed	52 (23)	54 (15)	
Missing	1 (0)	4 (1)	
*Workplace size*			.006
<50 employees	95 (43)	114 (32)	
50–249 employees	71 (32)	120 (33)	
≥250 employees	55 (25)	129 (35)	
Missing	1 (0)	1 (0)	
*Industry sector*			.023
Manufacturing	58 (26)	74 (20)	
Health and social services	27 (12)	74 (20)	
Wholesale and retail trade	23 (10)	24 (7)	
Public administration	16 (7)	26 (7)	
Construction and real estate	25 (12)	25 (7)	
Education, information, communication	21 (10)	45 (13)	
Other industry	50 (22)	95 (26)	
Missing	2 (1)	1 (0)	
*Occupational health care agreement*			.019
Preventive and medical services	177 (80)	268 (74)	
Preventive services only	36 (16)	52 (14)	
Don’t know	9 (4)	38 (11)	
Prefer not to say	0 (0)	5 (1)	
Missing	0 (0)	1 (0)	

*Note*. Responses marked as “Other” or “Prefer not to say” were treated as missing values in the analysis. *p* < .05 was considered statistically significant. The ISCED classification is based on UNESCO’s International Standard Classification of Education (UNESCO Institute for Statistics, 2024).

a Lower education degree = Basic/elementary school, vocational/technical/commercial school, upper secondary school, institute-level education (ISCED 1–4).

b High education degree = University of applied sciences, university/higher education (bachelor’s, master’s, doctoral degrees; ISCED 5–8).

Respondents rated collaboration with OHC on a scale from 0 to 10 (Table 3). The average rating given by OS managers was 7.76, while the average rating from OS representatives was 7.17 (*p* < .0001). Additionally, 39% of OS managers rated the collaboration as excellent (score 9–10), compared to 22% of OS representatives.

**Table 3.** Ratings of Collaboration With Occupational Health Care Among Occupational Safety Managers (*n* = 216) and Representatives (*n* = 352)

Rating of collaboration with occupational health care	Mean (95 % Cl)	*p*	Poor (0–5) %	Moderate (6–7) %	Good (8) %	Excellent (9–10) %
Occupational safety managers	7.76 [7.52, 7.99]	<.001	12	16	33	39
Occupational safety representatives	7.17 [6.97, 7.36]		18	28	32	22

*Note*. Ratings were based on a 0 to 10 scale, where 0 = very poor and 10 = excellent collaboration. Percentages represent distribution across rating categories. *p*-Value (Mann–Whitney *U*) compares mean ratings between groups and *p*-values <.05 are statistically significant.

**Figure 1.**
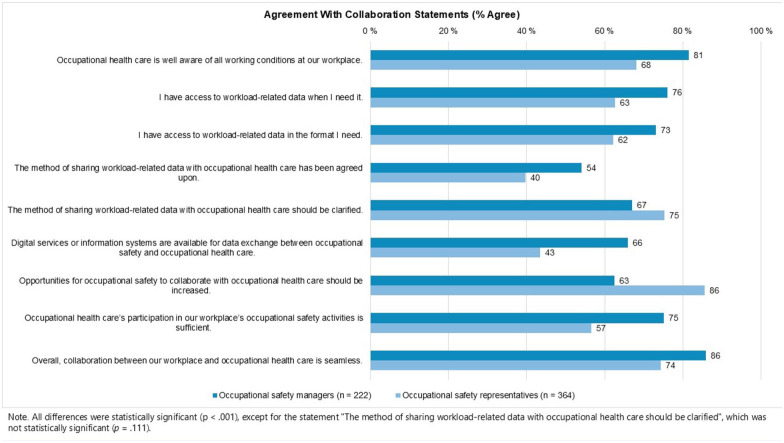
Occupational safety managers’ (*n* = 222) and representatives’ (*n* = 364) agreement with statements regarding collaboration with occupational health care. Values represent the percentage of respondents who agreed with each statement.

Respondents’ views on collaboration with OHC were also assessed using several attitude statements (Figure 1). Among OS managers (*n* = 222), 86% perceived the collaboration as seamless, whereas the corresponding proportion among OS representatives (*n* = 364) was 74% (*p* < .001). Furthermore, 86% of OS representatives believed that opportunities for collaboration between OS and OHC should be increased, compared to 63% of OS managers (*p* < .001). In contrast, the difference in views regarding the need to clarify the method of sharing workload-related data was smaller: 75% of OS managers and 67% of OS representatives agreed with the statement (*p* = .027).

### Factors Associated With Perceived Seamless Collaboration

As shown in Table 4, among OS managers, those working full-time in OS tasks rated seamless collaboration lower (mean = 2.7226) than those working part-time (mean = 2.8803) or on an as-needed basis (mean = 2.5050). In contrast, among OS representatives, full-time workers rated collaboration more positively (mean = 2.6461) than those working part-time (mean = 2.4510) or on an as-needed basis (mean = 2.2907).

**Table 4.** Associations Between Background Variables and Perceived Seamless Collaboration Among Occupational Safety Managers (*n* = 222) and Representatives (*n* = 364)

Variable	Occupational safety managers			Occupational safety representatives		
	Mean	Median	*p*	Mean	Median	p
Gender^a^						
Male	2.8460	2.8889	.363	2.5299	2.5556	.033
Female	2.7752	2.8889		2.4193	2.4444	
Age						
<40 years	2.8510	3.000	.989	2.3733	2.4444	.311
40–49 years	2.8443	2.7778		2.4683	2.5556	
50–59 years	2.8266	2.8889		2.5506	2.5556	
≥60 years	2.8270	3.0000		2.4611	2.4444	
Education^a^						
Low education degree (ISCED 1–4)	2.7560	2.8333	.562	2.4686	2.5556	.931
High education degree (ISCED 5–8)	2.8149	2.8889		2.4730	2.5556	
Work experience^a^						
≤20 years	2.8053	2.8889	.858	2.4621	2.5556	.744
>20 years	2.8020	2.8889		2.4794	2.5556	
Occupational safety experience^b^						
<5 years	2.7417	2.7778	.416	2.4346	2.4444	.616
5–10 years	2.8461	3.0000		2.5012	2.5556	
>10 years	2.8295	2.8889		2.5084	2.5556	
Working time in occupational safety^b^						
Full-time	2.7226	2.5556	.033	2.6461	2.7222	.002
Part-time	2.8803	3.000		2.4510	2.5556	
As needed	2.5050	2.6667		2.2907	2.2222	
Workplace size^b^						
<50 employees	2.8327	2.8889	.331	2.4555	2.4444	.191
50–249 employees	2.7944	2.8889		2.4163	2.4444	
≥250 employees	2.7608	2.7222		2.5350	2.6667	
Industry sector^b^						
Manufacturing	2.7998	2.8889	.707	2.5790	2.5556	.510
Health and social services	2.6163	2.7778		2.4327	2.5556	
Wholesale and retail trade	2.7343	2.7778		2.4769	2.4444	
Public administration	2.9676	3.1111		2.4017	2.3333	
Construction and real estate	2.8200	3.0000		2.5550	2.5556	
Education and information/communication	2.8360	2.8889		2.3735	2.333	
Other sectors	2.8755	2.8889		2.4634	2.5556	
OHC agreement^b^						
Preventive and medical services	2.8509	2.8889	.014	2.5186	2.5556	.007
Preventive services only	2.5444	2.6875		2.2299	2.2222	
Don’t know	2.8287	2.7778		2.3355	2.3333	

*Note*. Higher scores indicate more seamless collaboration. *p*-Values <.05 are statistically significant.

a Mann-Whitney (U).

b Kruskall-Wallis (H).

Additionally, those with an OHC agreement covering both preventive and medical care services rated the collaboration more positively (OS managers: mean = 2.8509; OS representatives: mean = 2.5186) than those with preventive services only (OS managers: mean = 2.5444; OS representatives: mean = 2.2299). Among OS representatives, gender was also associated with the experience of seamless collaboration: men (OS managers: mean = 2.8460; OS representatives: mean = 2.5299) rated the collaboration significantly more positively than women (OS managers: mean = 2.7752; OS representatives: mean = 2.4193).

The association between individual attitude statements and seamless collaboration was examined using Spearman’s rank correlation coefficient (Table 5). Based on responses from OS managers and representatives, the strongest association was found in OHC participation in workplace OS activities (*r* = .782). The availability of workload-related data was also a significant factor: both timely availability (*r* = .771) and content-appropriate format (*r* = .764) were strongly associated with the experience of seamless collaboration. Furthermore, having an agreed method for sharing data with OHC was clearly associated with more seamless collaboration (*r* = .765).

**Table 5.** Spearman’s Rank Correlation Coefficients Between the Sum Variable and Attitudinal Statements on Collaboration (*n* = 547)

Attitudinal statement (*n* = 547)	*r*
Occupational health care is well aware of all working conditions at our workplace.	.676
I have access to workload-related data when I need it.	.771
I have access to workload-related data in the format I need.	.764
The method of sharing workload-related data occupational health care has been agreed upon.	.765
The method of sharing workload-related data with occupational health care should be clarified.*	.564
Digital services or information systems are available for data exchange between occupational safety and occupational health care.	.523
Opportunities for occupational safety to collaborate with occupational health care should be increased.*	.576
Occupational health care’s participation in workplace’s occupational safety activities is sufficient.	.782
Overall, collaboration between workplace and occupational health care is seamless.	.721

*Note*. All correlations are Spearman’s rank-order coefficients. Items marked with * were reverse-coded. All *p*-values <.001.

## Discussion

The study examined the perspectives of OS managers and OS representatives on collaboration with OHC. The results revealed several factors associated with seamless collaboration, which may serve as focal points for strengthening cooperation.

OS managers rated collaboration with OHC as more seamless than OS representatives. They more often reported having sufficient access to information on workload factors, that OHC had a good understanding of workplace conditions, and that collaboration was based on clearly agreed practices. They also more frequently had access to digital services or information systems for data exchange, which may relate to their organizational position or stronger role in collaborative structures, such as being part of the workplace management team.

Although many OS managers perceived collaboration with OHC as excellent, a notable proportion also expressed a desire for increased collaboration. This apparent contradiction may reflect an understanding that, while current collaboration is adequate in quality, it could be more regular or proactive.

In contrast, OS representatives more often felt that collaboration and information exchange with OHC should be increased and that OHC’s participation in OS activities was insufficient. This more critical assessment may reflect their role as employee representatives which involves encountering more concerns and shortcomings related to working conditions. It may also indicate that their participation in collaboration does not always occur as intended. Particularly, representatives with low education degrees (ISCED 1–4) or those working in smaller workplaces rated collaboration as less seamless. It is evident that effective collaboration among OS and OHC requires not only a shared knowledge base but also jointly defined goals, clear communication practices, and agreed-upon procedures (Halonen et al., 2017; Paulsson et al., 2020; Pesonen et al., 2019). A well-conducted workplace survey and jointly planned activities serve as a strong foundation for such collaboration.

In addition, male OS representatives rated collaboration more positively than female representatives. This gender difference may reflect varying expectations of collaboration, such as differences in how communication, participation, or support from OHC are perceived or valued. However, further research is needed to explore these differences in more depth.

According to the results, seamless collaboration was especially influenced by the length of OS experience and the comprehensiveness of the OHC agreement. OHC participation in workplace OS activities and the availability of workload-related data were also strongly associated with more seamless collaboration. Respondents with longer OS experience and those whose OHC agreement included both preventive and medical services rated collaboration more positively on average. These factors were more common among OS managers, which may partly explain their more favorable assessments.

The results also revealed that respondents who perform OS tasks as needed rated collaboration with OHC as the weakest, compared to those working part-time or full-time. This finding was consistent among both OS managers and OS representatives suggesting that sporadic involvement in OS tasks may diminish positive experiences of collaboration with OHC. As previous research has shown, effective collaboration requires regular interaction and shared goals (Halonen et al., 2017; Pesonen et al., 2019) even in small workplaces (Pesonen et al., 2025). These elements should be ensured regardless of the amount of working time allocated to OS tasks.

The industry sector also influenced perceptions of collaboration. Respondents working in the public sector and in education rated collaboration less positively than those, for example, in the industrial sector. This may reflect more established collaboration channels, including digital systems in larger industrial workplaces. Additionally, in workplaces without traditional exposure risks, collaboration may be less frequent, as the focus shifts more toward psychosocial workload factors, even though ideally, this should not be the case.

In the future, technology may increasingly support collaboration between OS and OHC. AI-based sensors and wearable devices offer the potential to monitor the environment in real time and detect hazards (Dodoo et al., 2024) which could enhance data exchange and proactive measures. However, effective use of technology requires clear, jointly agreed procedures and division of responsibilities. Ensuring data protection remains essential to maintain trust between OHC, OS, and other workplace actors (Fiegler-Rudol et al., 2025; Paulsson et al., 2020; Sallinen, 2024).

The findings of this study reinforce previous evidence that collaboration between OS and OHC is a key factor in promoting workplace health and safety (Halonen et al., 2017; Lydell et al., 2017; Schmidt et al., 2016). However, collaboration is not always guided by clear objectives, and its effectiveness is often evaluated based on costs or sickness absences rather than improvements in working conditions (Paulsson et al., 2020). OHC could strengthen its ability to demonstrate the impact of preventive work and focus more on preventing workplace hazards rather than providing individual-level support.

Previous studies have also shown that OHC professionals value the opportunity to exchange information with employers about working conditions, occupational safety, and sickness absences at the group level, so that the information can be used for workplace development (Nissinen et al., 2021a, 2021b). However, workload factors are not always sufficiently identified (Majuri et al., 2024) which weakens the effectiveness of collaboration. Therefore, improving collaboration requires changes that support openness, trust, and the setting of shared goals.

The results of this study indicate that seamless collaboration is particularly built on the availability of information on workload, clear procedures, and the active participation of OHC. The findings highlight the importance of effective information flow and inclusive cooperation in the interaction between the workplace and OHC.

### Strengths and Limitations

This study was a cross-sectional survey aimed at examining the experiences of workplace OS actors (OS managers and OS representatives) regarding collaboration with OHC, and at addressing a knowledge gap identified in previous research.

A key strength of the study lies in its timeliness and broad sample, which included both OS managers and OS representatives from various sectors and organizations of different sizes. This enabled a multifaceted perspective on collaboration with OHC and revealed significant differences between the roles. The study also employed statistically robust methods, such as non-parametric tests and the construction of sum variable, which enhanced the precision of the analysis. Based on Cronbach’s alpha coefficients, the internal consistency of the sum variable was good, supporting the reliability of the measure.

Another notable strength was the study’s ability to identify factors influencing seamless collaboration, such as the comprehensiveness of the OHC agreement, OS experience, and the availability of digital tools. The study also made a valuable contribution by examining multiple dimensions of collaboration. For example, the availability and format of workload-related information, the use of digital systems, and OHC participation in OS activities formed a clearly operationalized framework that supports future research.

An additional strength was the development of a questionnaire specifically for this study. As no validated instruments were available on the topic, the questionnaire enabled the collection of targeted and contextually relevant data. Its design was informed by previous literature and the researchers’ expertise in OS–OHC collaboration, supporting its content validity.

However, the study also has limitations. The response rate was relatively low (15% for OS managers and 20% for OS representatives), which may affect the generalizability of the findings. It is possible that those who responded had particularly positive or critical experiences of collaboration. Furthermore, the study relied on self-assessment, which may introduce individual interpretation bias.

The use of a self-developed instrument also presents limitations in terms of measurement validity and reproducibility. Although internal consistency was good, structural validity was not assessed, which may limit the generalizability and comparability of the results with other studies. However, the study provides a valuable opportunity to preliminarily evaluate the questionnaire’s performance in a real-world setting. The high internal consistency supports its reliability, and the findings may contribute to the development of structured tools for assessing OS–OHC collaboration.

While the study revealed significant differences between OS actors, it did not explore in depth the organizational practices or structures that might explain these differences or shape overall views on collaboration.

## Conclusions

The study confirms that seamless collaboration between OHC and OS requires clear procedures, up-to-date information exchange, and the active participation of all parties involved. In particular, the availability of information related to workload factors and the participation of OHC in OS activities were key elements supporting successful collaboration. Collaboration can be improved through regular meetings and by ensuring equal access to information. Additionally, OHC can enhance information availability by providing more analyzed and proactive data on the workplace’s health and safety situation, as well as by developing its reporting practices for OS. In the future, qualitative research methods are also needed to deepen the understanding of the underlying factors influencing collaboration.

### Implications for Occupational Health Practice

Identifying workload factors related to working conditions and understanding their effects is essential for the effectiveness of OHC activities. OHC professionals have a responsibility to highlight these factors and communicate them clearly to workplace management and OS actors. Their expertise is also crucial in identifying work-related resources, which can serve as protective factors against occupational health and work ability risks. OHC is expected to engage in active dialogue and to translate observations into concrete recommendations that support the development of working conditions.

The effectiveness of collaboration is strengthened when OHC meets regularly with OS actors, shares the aggregate results of health examinations and workplace surveys, and supports the targeting of measures based on up-to-date information. Clear and structured reporting helps OS utilize the information provided by OHC in decision-making and in prioritizing development actions.

Demonstrating the impact of preventive occupational health work can be challenging. Impacts such as reduced sickness absences or improved working conditions are not always immediately visible. However, regular interaction between OHC and OS actors can help make the benefits of collaboration more tangible. When OS actors have access to up-to-date and clearly structured information, they are better able to justify necessary measures and promote healthier and safer working conditions within their organization.

As working life evolves, OHC professionals must maintain and update their competence in identifying workload and resource factors and assess the impact of their actions as part of a comprehensive approach to promoting workplace well-being.

**Applying Research to Occupational Health Practice**

The findings of this study provide concrete insights for developing collaboration practices between OS and OHC. OHC should pay particular attention to the timely and clear communication of workload-related factors and actively participate in OS activities, as these were the factors most strongly associated with the experience of seamless collaboration. Furthermore, the role of OHC as a coordinator of collaboration becomes especially important in situations where there are differences between OS managers and OS representatives in terms of access to information or opportunities for participation.
